# Evaluation of the immunogenicity and efficacy of a chimeric OMP25–OMP31 antigen in BALB/c mice

**DOI:** 10.1002/vms3.537

**Published:** 2021-05-31

**Authors:** Yahya Mohammadi

**Affiliations:** ^1^ Department of Animal Science, Faculty of agriculture Ilam University Ilam Iran

**Keywords:** antigenicity, Brucella, Brucellosis, OMP25, OMP31

## Abstract

Brucellosis is a zoonotic disease causes by *Brucella* bacteria. So far, there is not any efficient treatment for this infectious disease in animals, although subunit vaccines can be a safe alternative. To this aim, we have designed a new chimeric OMP25‐OMP31 antigen formulated in Chitosan nanoparticles and studied its protective efficiency in vivo. OMP25–OMP31 was produced using spliced overlap extension by polymerase chain reaction and the 3D protein structure and antigenic ability were predicted using computational tools. In addition, the humoural and cellular immune responses were measured by ELISA in six different experimental groups. The immune response showed chimeric rOMP25–OMP31 antigen‐induced higher titers of IFN‐γ and TNF‐α cytokines, while the lowest amount of IL‐4 was dedicated to itself. Also, rOMP25–OMP31 stimulated higher titer of IgG than individual injection of rOMP25 and rOMP31 treatments and the cell proliferation assay demonstrated the vaccination with rOMP25–OMP31 elicits a vigorous antigen‐specific cell proliferative. In addition, the challenge experiment showed immunised mic stimulated a higher level of protection than negative controls. Overall, the results of rOMP25–OMP31 could be promising for considering chimeric constructs as a feasible vaccine candidate for further investigations against brucellosis.

## Graphical abstract

Chimeric antigen induced better immune response.

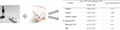

## 1 INTRODUCTION

*Brucella melitensis* as one of the *Brucella* bacteria strains leads to the most cases of human brucellosis which is found in sheep and goat (Mohamed Zahidi et al., 2017; Tadepalli et al., 2016). This infection is transmitted to humans through direct contact or the consumption of unpasteurized products from infected animals (Wareth et al., 2014). Although there is not any efficient treatment for brucellosis in animals, several studies have recently been introduced a few number of subunit vaccine candidate with significant protective in vivo (Cassataro et al., 2005; Clausse et al., 2013; Pasquevich et al., 2009; Sekhavati, Majidzadeh Heravi, et al., 2015; Tab ynov et al., 2014; Yang et al., 2011). OMP25 and OMP31 are two of immunogenic candidates which are able to provide appropriate immunity and protection against *Brucella* infection (Abbassi‐Daloii et al., 2019; Clausse et al., 2014; Diaz et al., 2013; Shojaei et al., 2018; Yousefi et al., 2018).

In this study, we assume that designing a new chimeric construction containing two major OMP25 or OMP31 *Brucella* antigens formulated in Chitosan nanoparticles (CS‐NPs) would improve immunogenicity. To keep a stable distance between two proteins with independent functions, the recombinant proteins were linked using an EAAAKEAAAK rigid linker (Chen et al., 2013). The challenging results showed injection of chimeric rOMP25–OMP31 was not as efficient as live attenuated vaccine, but it could enhance immunity compare to individual injection of rOMP25 and rOMP31.

## 2 MATERIALS AND METHODS

### 2.1 Characteristics of OMP25‐OMP31

OMP25 (642 bp) and OMP31 (723 bp) antigens extracted from *B. melitensis Rev1* strain using specific primers and their products were confirmed by gel electrophoresis and western blotting as described previously by Tahmoorespur et al., 2016; Yousefi et al., 2016; Yousefi et al., 2016. The EAAAKEAAAK rigid linker was used to keep a stable distance between two proteins with independent functions (Chen et al., 2013). The chimeric rOMP25–OMP31 antigen was produced using spliced overlap extension by polymerase chain reaction (SOE‐PCR) approach (Sekhavati, Tahmoorespur, et al., 2015). *Escherichia coli* TOP10F` and BL21 (DE3) were used as cloning and expression hosts, respectively. Moreover, I‐TASSER and VaxiJen 2.0 tools were used to assess the structural and antigenic ability of the rOMP25–OMP31 construct, respectively (Doytchinova & Flower, 2007; Zhang, 2008).

Isopropyl‐β‐D‐thiogalactopyranoside (IPTG) of 1 mM was used to induce the production of recombinant proteins. They were dialysed at 4°C overnight and columns were washed twice by cold phosphate‐buffered saline (PBS). The dialysed proteins were mixed with nickel‐charged affinity resins and purified through Ni‐agarose (Thermo) according to the manufacturer's protocol. All proteins were devoid of the periplasmic part of the BL21 (DE3) bacterium which may contain bacterial lipopolysaccharide (LPS).

### 2.2 Vaccine preparation

The BALB/c mice (6 weeks old, Female) were randomly classified into six experimental groups (5 mice/group). All animal housing and experiments which were done using the animals in this study are in agreement with the Ethical Principles for Animal Research established by Ilam University, Ilam, Iran.

Each experimental group was injected intraperitoneally (IP) three times (each time 30 µg of recombinant proteins) with 2 weeks interval (days: 0, 15 and 30). To remove any interference effect of PBS and self‐expressed pET‐32a^(+)^ vector on the immune responses, these groups were considered as negative controls. Also, a dose of live attenuated vaccine *B. melitensis* Rev1 (1–4 × 10^9^ CFU/mice) was injected as a positive control group. All injections were emulsified in 50 μl of CS‐NPs (Sigma), and PBS was added to each vaccine mixture to a final volume of 300 μl. Yousefi et al., (2019) have investigated the immune response between aluminium hydroxide, incomplete Freund and CS‐NP, and they have found CS‐NPs could enhance the immune response more than other adjuvants (Yousefi et al., 2019).

### 2.3 Experimental procedure

Serum was collected by centrifugation (3,000 g, 20 min) from whole blood 15 days after the last injection. Humoural immune response was measured by coating 1 µg/ml of each purified recombinant protein or 1 × 10^8^ CFU of Rev1 strain of *B*. *melitensis* in 96‐well plates (Nunc, Naperville, IL) and incubated for 24 hr at 37°C. Then, wells were washed three times with PBS and 0.05% Tween 20 (TPBS), 5% skimmed milk in PBS used for blocking at 37°C for 1 hr. Plates were incubated with serial dilutions of mouse sera (1:100–1:10,000) for 2 hr at 37°C. Then, 100 µl of 1:10,000 dilution of anti‐mouse IgG–Horseradish Peroxidase (HRP)‐conjugate antibody (Sigma, USA) was added to each well and incubated at 37°C for 2 hr. Plates were washed five times and incubated for 15 min with 100 µl of 3,3’,5,5’‐tetramethyl‐benzidine (TMB) substrate in the dark and the reaction stopped using 2N H_2_SO_4_. Colour quality was quantified at OD_405_ nm. In addition, 100 µl of 1:4,000 dilution of goat anti‐mouse IgG1 and IgG2a antibodies were used to determine the Th1 or Th2 immune response balance.

To measure interferon gamma (IFN‐γ), tumour necrosis factor‐alpha (TNF‐α) and interleukin‐4 (IL‐4) responses, the spleens of sacrificed mice were separated and homogenised in 10 ml PBS containing 5 mM ethylenediamine‐tetraacetic acid (PBS‐EDTA) on ice. Then, the mononuclear cells, which were isolated by centrifuging, were cultured in RPMI 1,640 at 37°C in 5% CO_2_, to adjust and coat a total number of 4 × 10^6^ cells in 24‐well plates. Antigen recall was performed by adding 10 μg/ml of each recombinant proteins to wells and incubated for 48 hr at 37°C in 5% CO_2_. Finally, cell culture supernatant was collected by centrifuging (300 g,10 min) to determine cellular immunity.

In addition, to evaluate the stimulation index as a lymphocyte proliferation assay, the stimulated wells by recombinant proteins were treated by 20 µl MTT (3‐[4,5dimethylthiazole‐2yl]‐2,5 diphenyl tetrazolium bromide, 5 mg/ml) for 4 hr, followed by adding 100 µl of dimethyl sulfoxide (DMSO) and incubation for 1 hr. Absorbance was measured using a spectrophotometric plate reader at 590 nm.

### 2.4 Protection experiment

Four weeks after the last immunisation, mice were challenged through IP injection of 1 × 10^4^ CFU of *B. melitensis* 16 M. Infected animals were sacrificed by cervical dislocation 15 days after being challenged and their spleens were separated and homogenised in 1 ml of PBS, then each 10‐fold serial dilution was seeded on *Brucella* agar plates and incubated for 3 days at 37°C in 5% CO_2_. The content of bacteria in each spleen was counted and represented by mean log_10_ CFU ± *SD* of treatments. The protection units were determined by subtracting the log CFU of each immunised group from the PBS control group.

### 2.5 Statistics

GraphPad Prism v6.07 software (GraphPad Software Inc.) was used to measure parameters. One‐way analysis of variance (ANOVA), followed by Tukey's *post hoc* test was considered to compare experimental groups, and significant comparison was selected based on *p‐value* < 0.05. All values were indicated as mean ± *SD*.

## 3 RESULTS

### 3.1 Structural features of OMP25_OMP31 construct

**FIGURE 1:**
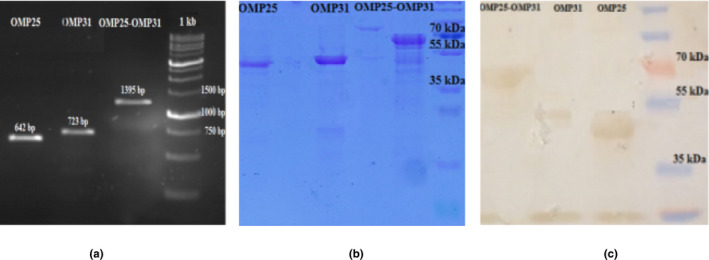
(a) Amplicons of OMP25, OMP31 and chimeric OMP25–OMP31 antigens. (b) Purified OMP25, OMP31 and chimeric OMP25‐OMP31 proteins were extracted by Ni‐NTA affinity chromatography with ~43, ~49 and ~67 kDa, respectively. (c) Western blot confirmation of recombinant proteins by anti‐poly‐Histidine‐HRP antibody

The quality and identity of the chimeric rOMP25‐rOMP31 protein with ~67 kDa molecular mass was measured using SDS‐PAGE (10%) and western blotting (Sigma) (Figure 1).

**FIGURE 2:**
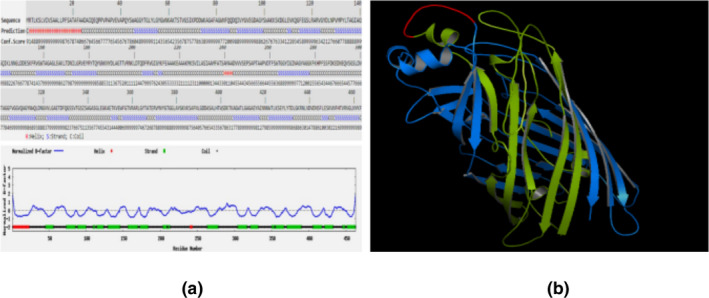
Predicted secondary and 3D structures of OMP25–OMP31. (a) Secondary structure: lines in different colours (red colour is a helix, the blue colour is strand and the black colour is coil) represent different secondary structures. Also, the blue line shows B‐factor. (b) 3D structure: the green, red and blue structures refer to OMP25 recombinant protein, linker and OMP31 recombinant protein, respectively

The secondary and 3D structural results showed the proportion of α helices, beta strand and random coils accounted for 5.41%, 43.19% and 51.4% of the secondary structure, respectively (Figure 2). Most of the amino acids showed a confident score of 8 or 9, as a high score means more confident of secondary structure (Figure 2). The blue line in Figure 2a shows B‐factor indicates the extent of the inherent thermal mobility of residues in proteins, and the negative values mean that the residue is relatively more stable in the structure (Yang et al., 2016) like most of the residues in our chimeric construct. Also, the protein structure showed that there is a fixed distance between two proteins indicating the efficiency of the linker (Figure 2b). The antigenic ability of the rOMP25–OMP31 protein was determined 0.75, whereas rOMP25 and rOMP31 individually showed antigenic scores 0.82 and 0.67, respectively.

### 3.2 Humoural and cellular immune responses

**FIGURE 3:**
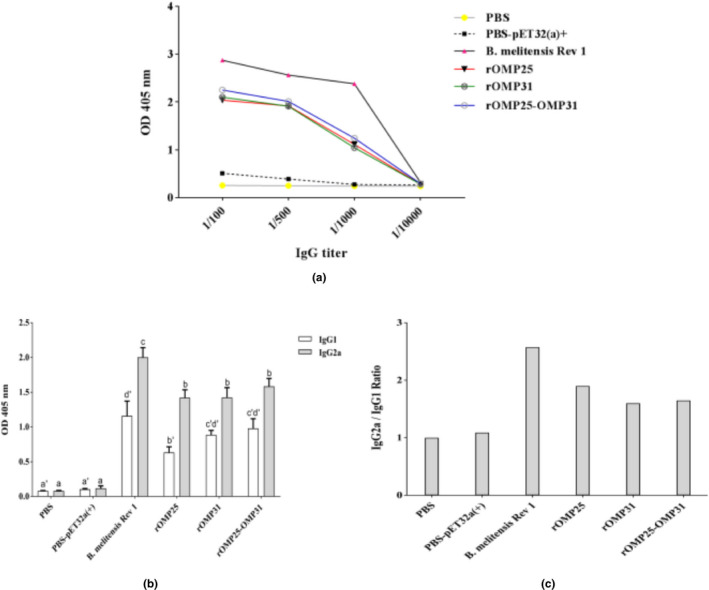
Kinetics production of antibody. a) Total antibody titer for each treatment. b) IgG1 and IgG2a responses in immunised mice. c) The ratio of IgG2a to IgG1 in immunised mice. Levels of each antibody were measured at OD_405_ nm with an ELISA reader. PBS and PBS‐pET‐32a ^(+)^ refer to negative control groups. Live attenuated vaccine *B. melitensis* Rev1 refers to positive control groups. Each value represents the mean of triplicates ± *SD* of antibody responses from five samples. Different letters indicate statistically significant differences between experimental groups that carried out using Tukey's test (*p* < .05)

Immunoglobulin G (IgG) was assessed by indirect ELISA to measure humoural immunity. Total antibody response showed immunisation with different treatments improved level of antibody amount compared to the negative control groups, although it was still lower than the positive control group (Figure 3a). The rOMP25–OMP31 showed chimeric construct could enhance IgG level compared to individual OMP25 and OMP31 injections (Figure 3a). Figure 3 shows higher levels of both IgG1 (Th2) and IgG2a (Th1) antibodies in immunised groups in comparison to the negative control groups (*p* < .05). IgG1 results showed that there was no statistically significant difference between rOMP31, rOMP25–OMP31 and positive control groups, whereas the titer of IgG2a antibody between rOMP25, rOMP31 and rOMP25–OMP31 was statistically similar and lower than the positive control group (*p* < .05, Figure 3b). Although the average ratio of IgG2a/IgG1 was ~1.59 indicating a strong switch from Th2 to Th1 immune response, rOMP25 revealed the highest ratio and rOMP25–OMP31 was higher than rOMP31 (Figure 3c).

**FIGURE 4:**
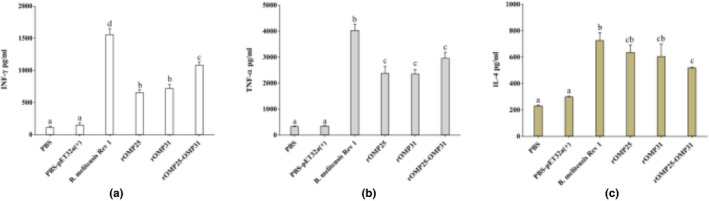
Determination of cytokine responses in spleen cells of immunised mice. (a) IFN‐γ response in immunised mice with different recombinant proteins. (b) TNF‐α response in immunised mice with different recombinant proteins. (c) IL‐4 response in immunised mice with different recombinant proteins. Levels of each cytokine were quantified (pg/mL) by ELISA. PBS and PBS‐pET‐32a ^(+)^ refer to negative control groups. Live attenuated vaccine *B. melitensis* Rev1 refers to positive control groups. Each value represents the mean of triplicates ± *SD* of antibody responses from five samples. Different letters indicate statistically significant differences between experimental groups that carried out using Tukey's test (*p* < .05)

Sandwich ELISA strategy was used to measure cytokine secretions (Figure 4). Chimeric protein statistically induced higher levels of INF‐γ compared to rOMP25 and rOMP31 proteins. However, there were not statistically significant differences for TNF‐α and IL‐4 between chimeric and individual proteins (*p* < .05, Figure 4).

**FIGURE 5:**
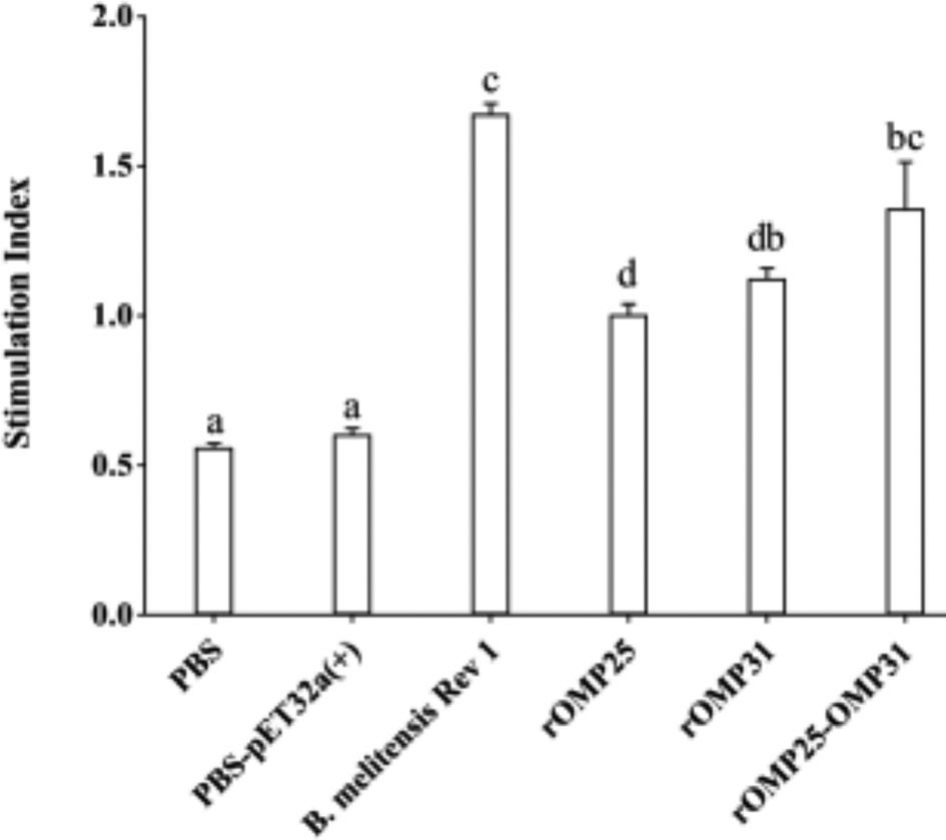
Lymphocyte proliferation responses of the experimental groups after in vitro antigen recall. PBS and PBS‐pET‐32a ^(+)^ refer to negative control groups. Live attenuated vaccine *B. melitensis* Rev1 refers to positive control groups. Each value represents the mean of triplicates ± *SD* of antibody responses from five samples. Different letters indicate statistically significant differences between experimental groups that carried out using Tukey's test (*p* < .05).

Also, the lymphocyte proliferation result revealed that chimeric protein had a higher titer than individual groups and it showed statistically a similar titer as a positive control group (Figure 5).

### 3.3 Protection against B. Melitensis

The protective efficiency was calculated based on the number of live bacteria in the spleen of immunised mice using recombinant proteins and Rev 1 compared to the negative control groups (PBS groups). As shown in Table 1, all immunised mice conferred protection against *B. melitensis* 16 M than negative control groups (*p* < .05). The rOMP25–OMP31 vaccine represented 2.16 log unit of protection against *B. melitensis* 16 M, it was higher than rOMP25 and rOMP31 vaccines. However, it is still lower than the live attenuated *B. melitensis* Rev1 with 3.07 log unit of protection.

**TABLE 1:** Protective efficiency of immunised mice against *B. melitensis* 16 M. The data are represented as mean log_10_ CFU ± *SD*

Vaccine	Log_10_ CFU of *B. melitensis* 16 M (mean ± *SD*)	Log_10_ protection unit
rOMP25	5.08 ± 0.41^b^	1.27
rOMP31	4.46 ± 0.67^bc^	1.89
rOMP25–OMP31	4.19 ± 0.45^c^	2.16
*B. melitensis* Rev 1	3.28 ± 0.38^d^	3.07
PBS + pET−32a^(+)^	6.23 ± 0.13^a^	0.12
PBS	6.35 ± 0.34^a^	0

Different letters indicate statistically significant differences between experimental groups that carried out using Tukey's test (*p* < .05).

## 4 DISCUSSION

Brucellosis or Mediterranean fever affects a wide range of animals and leads to serious nervous and movement disorders in humans (Kim et al., 2017). Although the humoural immune response is important for protection against brucellosis, immunity mainly depends on the cellular response (Mansoori & Pourmand, 2016; Pasquali et al., 2001). To date, the most efficient protection has been supplied by live attenuated vaccines stimulating strong cell‐mediated immunity (Sancho et al., 2014). However, those vaccines have some limitations including pathogenicity for humans, abortion in pregnant animals and interfering with the diagnostic tests (Cassataro et al., 2005). In recent decades, subunit vaccines are becoming promising candidates against *Brucella* infection due to less biohazardous, non‐infectious and non‐viable than live vaccines, though they cannot replicate the immunogenicity of later vaccines. To develop a new vaccine, two factors should be considered as follows: (a) selecting an immunogenic antigen with the potential to induce remarkable Th1 immunity and confer the high level of protection and (b) using a proper adjuvant to enhance vaccine efficacy (Clausse et al., 2014; Golshani & Buozari, 2017). In this study as part of an ongoing project, we assumed that the simultaneous injection of OMP25 and OMP31 as a chimeric construct probably induces higher immunogenicity, particularly in combination with CS‐NPs adjuvant.

The immune responses showed, rOMP25‐OMP31 construct induced higher titer of IFN‐γ cytokine, while there were no statistically significant differences for TNF‐α and IL‐4 cytokines between chimeric and individual recombinant proteins. In addition, individual injection of rOMP25 and rOMP31 showed statistically the same level of stimulation for all cytokines (Figure 4). These results could be endorsed by the antibody amounts witnessed a skew from IgG1 to IgG2a (particularly for rOMP25‐OMP31 than other recombinant proteins) in immunised mice, the IgG2a/IgG1 ratio with the average of ~1.59 indicating a strong bent of Th1 immune response which is important for secreting IFN‐γ. As IFN‐γ causes switching of Ig genes to IgG2a predominates and IL‐4 (Th2 cells) promotes Ig switching to IgG1 (Golding et al., 2001). In addition, the cell proliferative response of rOMP25–OMP31 group showed that the chimeric construct could be vital for controlling brucellosis by eliciting a vigorous antigen‐specific cellular response (Figure 5). Moreover, protection ability showed chimeric vaccine had a lower number of live bacteria in the splenocytes compared to negative and individual vaccines.

Our results were in agreement with Clausse et al., (2013), Estein et al., (2003) and Diaz et al., (2013) which showed the chimeric BLS‐OMP31 construct induced efficient humoural and cellular responses and conferred protection against *B*. *canis* and *B. ovis*. In addition, Yousefi et al., (2018) observed the chimeric OMP25‐BLS improved immunity compare to individual injections of OMP25 and BLS recombinant proteins. In another study, in vivo immunisation of L7/L12‐TOmp31 recombinant protein provided significant immunity and protection against *B. abortus* and *B*. *melitensis* through inducing IgG2a response, IFN‐γ production and T‐cell proliferation (Golshani et al., 2015).

Abbassi‐Daloii et al., (2019) studied the impact of several concentrations of recombinant OMP25 and OMP31 proteins as univalent and divalent injections on immunity, they found divalent injection of OMP25 and OMP31 (rOMP25+rOMP31) with equal concentrations showed higher immune responses than other groups. Also, Tadepalli et al., (2016) studied protection efficiency of univalent rOmp19, rP39 and divalent rOmp19+rP39 injections and realised that immunised mice with rOmp19+rP39 induced significantly higher cytokines and IgG2a antibody responses than univalent injected groups. Also, other studies have shown higher levels of cellular immunity, as well as, IgG2a and IgG1 antibody amounts associated with protection and immunity against *Brucella* that can be helpful to develop a feasible vaccine (Abbassi‐Daloii et al., 2018; Al‐Mariri, 2010; Cassataro et al., 2005; Clapp et al., 2011).

## 5 CONCLUSIONS

However, rOMP25–OMP31 showed slightly better immune responses (particularly based on IFN‐γ cytokine and protective efficiency) than individual injection of rOMP25 and rOMP31 proteins, it can be a promising candidate for further investigations or considering it beside other antigens as one vaccine construct to enhance immunity.

## CONFLICT OF INTEREST

No conflict of interest.

## AUTHOR CONTRIBUTION

**Yahya mohammadi:** Conceptualization; Data curation; Formal analysis; Funding acquisition; Investigation; Methodology; Project administration; Resources; Supervision; Writing‐original draft; Writing‐review & editing.

### PEER REVIEW

The peer review history for this article is available at https://publons.com/publon/10.1002/vms3.537.

## Data Availability

Data will be available upon request.
